# The New Unemployment Insurance Act

**Published:** 1916-09-16

**Authors:** 


### NATIONAL INSURANCE ACT DEVELOPMENTS.

### The New Unemployment Insurance Act.

It is seldom that we deal with unemployment insurance topics in our National Insurance articles, for the effect of unemployment insurance upon public health is much less obvious, even if it is not actually less, than that which is, or should be, exerted by the complex organisation known as National Health Insurance. Nevertheless, it is an undoubted fact that regular employment is conducive to health, provided the occupation is in itself of a reasonably healthy character. There are occupations, of course, in which the workers continually subject themselves to dangers to life and limb, either by accident or exposure, and there are others still in which the workers run the risk of contracting some special malady as a direct result of that employment. In these cases it would doubtless be correct to say that the particular employment was deleterious to health, and this would not mean that regularity or irregularity had any influence in the matter.

In general, however, it may be said that regularity in employment is beneficial to the health of the workers, for the regular habits of life engendered thereby, and the regular wages obtained therefrom, enable the workers to obtain regular and proper nourishment, and are therefore conducive to their general health and well-being. Unemployment brings with it not only the unsettlement and mental anxiety which is associated with finding new, congenial, and remunerative employment, but, unless specially provided for, it also entails the lack of immediate resources wherewith to obtain food and comforts usually obtainable when in work.

Common observation leads to the conclusion that the health of a workman in regular employment is far better, and his happiness far greater, than that of a man only casually employed, even though when in work the latter may make more money than the former.

### Labour Exchanges and Public Health.

In this sense, therefore, unemployment has a very material bearing upon the health of the community, and the labour exchanges, introduced some few years ago, have probably done a great deal, almost unknowingly, in the way of improving the public health. Yet in spite of labour exchanges, at which employers and workers can be brought into touch with each other's requirements, it is not possible to obviate entirely all unemployment. Fortunately, at the present time the great demands for munitions of all sorts—guns, ammunition, food, clothing, and all that is required to supply a Navy and Army of unprecedented magnitude—have reduced unemployment to a negligible quantity. It must not, however, be supposed that this state of affairs will continue indefinitely. On the contrary, the present industrial activity and boom in trade, being due to a special cause, and not being the result of any gradual economic development, are likely to be attended with grave results, which will be difficult to deal with, unless preparation be made in advance, when that special cause ceases to operate.

### Economic Problems after the War.

Upon the termination of the war, whether soon or far distant, we may look for an almost immediate cessation of the manufacture of instruments of destruction. This will set free for other purposes some millions of hands which to-day are occupied as fully as they can be. It may be that accumulated arrears of works of construction, in metals of all descriptions, will tend to retain in full employment for a considerable while—at least, until the Army has been disbanded—a large number of those who have entered the munition workshops during the present time of stress. In other directions it is easy to see that demands will arise to keep business and employment in full swing for a long while after peace is declared. Those, for instance, who are now engaged in the manufacture of khaki cloth, and the making of that cloth into army clothes, will, doubtless, be required to turn their attention to weaving other cloths and making other garments with which to clothe the returning millions from the war, to restore depleted stocks, and to provide the manufactured goods which will be required to rehabilitate our trade across the Atlantic and elsewhere. Yet, in spite of all these various uses to which the activities of munition workers—in the widest sense of that term—may be diverted, it may be anticipated that the months immediately following the declaration of peace will be a time of unrest and economic difficulty. Food prices will not have been restored to their pre-war figures, and, in consequence, unemployment—if there be any on a large scale—will tell more heavily against the workers than it did in former times. Moreover, it has to be borne in mind that upon discharge from the Navy or Army many of our gallant men will be faced with the difficulty of finding suitable work. Some, it is true, will have their old places kept open for them—it is to be hoped that the number will be considerable—others, unfortunately, even though their former employers would be willing to take them back, will be physically incapable of undertaking their customary duties. Even when a man is taken back by his former employer, it will most likely mean that another man, or a woman, who has been doing duty in his absence, will have to find work elsewhere. Again, although money is now flowing freely, it by no means follows that there will be the same amount of money for a long time after the war as there formerly was, for the support of industries connected with the manufacture and purveyance of luxuries. This will mean the shifting of workers from one class of employment to another, and in the process it is impossible to escape unemployment on more than what has been considered normal lines.

Preparation to be made now.

These are merely indications of the large and difficult problems that will, in the more or less distant future, have to be faced, and for which solutions will have to be found. If it is true that as a nation we were unprepared for war, that is no reason why we should be unprepared for the return of peace. After the war cannot be the same as before the war. The war has taught and is teaching the interdependence of each section of the community, and it is therefore necessary that all who are in the position of employers should begin even now to think out solutions to the problems, so far as they can be foreseen, that will materialise at the conclusion of the war.

The Example of the New Unemployment Insurance Act.

It is in these circumstances that we welcome, by way of example from the Government, the latest addition to the Statute-book in connection with national insurance—the new National Insurance (Part II.) (Munition Workers) Act.

The Act has hardly attracted the amount of attention that its merits warrant. It has probably been considered as an emergency measure, and has been passed over, except by those who are immediately concerned, as a matter of no great importance. Being of a non-contentious character, it passed through Parliament with a degree of smoothness which would have filled with envy those who were responsible for piloting the principal Act through the troubled Parliamentary debates through which it had to pass.

The object of the Act is to extend the provisions of unemployment insurance temporarily for the benefit of workers now employed on munitions. In other words, instead of those provisions of the Act of 1911 being restricted to the building and engineering trades, they are now to be extended to include those engaged in a number of other trades, in which there is at the moment unexampled prosperity. The list of trades to which the extension of the Act is to apply is set out in the first schedule, and includes: (1) the manufacture of ammunition, fireworks, and explosives; (2) the manufacture of chemicals, including oils, lubricants, soap, candles, paints, colours, and varnish; (3) the manufacture of metals and the manufacture or repair of metal goods; (4) the manufacture of rubber and goods made therefrom, (5) the manufacture of leather and leather goods; (6) the manufacture of bricks, cement, and artificial stone and other artificial building materials; (7) saw-milling, including machine woodwork, and the manufacture of wooden cases.

This is an impressive list, and it is estimated that one and a half million workers will be affected. The provision that is being made for them is this, that if unemployment should unfortunately overtake them, they will be entitled to claim the unemployment benefit, which should be of considerable value in helping to tide over the time until new work has been found.

The Act came into operation on September 4, as from which date contributions became payable. As at present drafted it is not designed to remain permanently in force. Its operation is limited to a period of five years from the commencement of the Act or three years after the termination of the present war, whichever may be the later. It will, of course, remain to be seen whether Parliament may not subsequently determine to extend unemployment insurance to these trades permanently; but in the meantime, as there were no means of ascertaining the liability that would devolve upon the Treasury in guaranteeing the solvency of the unemployment insurance fund so far as these newly accepted risks are concerned, it became necessary to impose some limit upon the time during which the Act is to operate.

The principal point to be noted, as it appears to us, is that advantage has been taken by the Government of a time of unprecedented employment and prosperity to pass a measure whose object is to accumulate a fund against a possible period of acute unemployment and economic depression. No adverse criticism has, so far as we are aware, been levelled at the Government for the step they have taken. It all goes to show that when the necessity is appreciated there is no difficulty in providing the necessary means for overcoming the difficulty.

Employers Concerned as well as the Government.

Insurance against unemployment, however, only partially mitigates the evil, and it is to be hoped that although employers are fully occupied with the problems concerning their immediate output, they will find some time for thought, and action—if necessary—in order that the period of reconstruction that is bound to ensue after the termination of the war shall be aggravated as little as possible by causes which a little forethought could have averted. In urging these considerations we feel that we are advocating a matter of the highest importance in the interests of the health and well-being of the community, and we doubt not that all who have the power will see that everything possible is done that can be done in the directions indicated.

